# Gene regulatory networks inference using a multi-GPU exhaustive search algorithm

**DOI:** 10.1186/1471-2105-14-S18-S5

**Published:** 2013-11-05

**Authors:** Fabrizio F Borelli, Raphael Y de Camargo, David C Martins, Luiz CS Rozante

**Affiliations:** 1 Center for Mathematics, Computing and Cognition, Federal University of ABC, Av. do Estados, 5001, Santo André -SP, Brazil

## Abstract

**Background:**

Gene regulatory networks (GRN) inference is an important bioinformatics problem in which the gene interactions need to be deduced from gene expression data, such as microarray data. Feature selection methods can be applied to this problem. A feature selection technique is composed by two parts: a search algorithm and a criterion function. Among the search algorithms already proposed, there is the exhaustive search where the best feature subset is returned, although its computational complexity is unfeasible in almost all situations. The objective of this work is the development of a low cost parallel solution based on GPU architectures for exhaustive search with a viable cost-benefit. We use CUDA™, a general purpose parallel programming platform that allows the usage of NVIDIA^® ^GPUs to solve complex problems in an efficient way.

**Results:**

We developed a parallel algorithm for GRN inference based on multiple GPU cards and obtained encouraging speedups (order of hundreds), when assuming that each target gene has two multivariate predictors. Also, experiments using single and multiple GPUs were performed, indicating that the speedup grows almost linearly with the number of GPUs.

**Conclusion:**

In this work, we present a proof of principle, showing that it is possible to parallelize the exhaustive search algorithm in GPUs with encouraging results. Although our focus in this paper is on the GRN inference problem, the exhaustive search technique based on GPU developed here can be applied (with minor adaptations) to other combinatorial problems.

## Background

The cell is a complex system where its activity is controlled by gene regulatory networks [1]. The mRNA concentration produced by each gene indirectly reflects its expression level. These concentrations can be an indication of the biological state of the cell, since they represent the proteins synthesized by ribosomes [2]. Thus, the biological processes studies can be based on the analysis of mRNA concentrations (expression levels) of the genes. DNA *microarrays *[3], SAGE (*Serial Analysis of Gene Expression*) [4] and RNA-Seq [5] are among the most common techniques to measure the expression level of thousands of genes at the same time.

A vast amount of transcriptome data has been provided by these large scale techniques, whose analysis requires efficient computational tools. In this context, the inference of gene regulatory networks (GRNs) aim to obtain the interactions among genes from gene expression data. Due to its relevance, several methods for GRN inference have been proposed, including Bayesian networks based [6,7], relevance networks [8], ARACNE (*Algorithm for the Reconstruction of Accurate Cellular NEtworks*) [9] CLR (*Context Likelihood of Relatedness*) [10], and SFFS-MCE (*Sequential Floating Forward Selection - Mean Conditional Entropy*) [11-13]. For reviews on this topic, the reader can be referred to [14-19].

Although many GRN inference methods are available, there are still challenges to overcome, such as noisy data, computational complexity and the curse of dimensionality (number of variables much larger than the number of available samples). Solutions based on high-performance computing are interesting when the objective is to infer GRNs with thousands of genes, although traditional platforms are expensive and difficult to maintain.

In this context, GPU (*Graphics Processing Unit*) for general purpose computing (GPGPU) is an emergent technology which allows to perform high-performance computing with relatively low cost [20,21]. CUDA (*Compute Unified Device Architecture*) is a programming platform which provides a parallel programming model allowing the NVIDIA GPU architectures to perform efficient general purpose computing.

The employment of GPUs to address the GRN inference problem is very recent though. Shi *et al*. proposed a parallelization scheme for GRN inference based on information-theoretic framework which involves matrices multiplication, optimizing the benefit obtained by applying GPU [22]. This method results in an approximation considering only pairwise relationships between genes, without taking into account the multivariate predictiveness nature of certain predictor genes with respect to the target genes. Here we present a GPU-based parallel exhaustive search algorithm, with mean conditional entropy as criterion function, for GRNs inference with two multivariate gene predictors per target gene. The gene network inference approach of the proposed algorithm is based on probabilistic gene networks [11], which displayed interesting results in obtaining the best predictor pairs for the considered target genes, given a data set with ternary values (-1,0,+1). We obtained speedups (six-core CPU was taken as reference) of 190 for ternary data samples and 260 for binary data samples when using 4 GPUs in networks with 8192 genes and almost linear increases in the speedup versus the number of GPUs. Consequently, using our algorithm, the exaustive search of predictor genes in GRNs can be performed in a reasonable amount of time.

The present paper is an extended version of the paper "Accelerating gene regulatory networks inference through GPU/CUDA programming" [23]. The main improvements found in this manuscript include: i - an improved version of the algorithm that works on multiple GPUs, instead of a single GPU; ii - a more complete description of the model used to infer gene networks from temporal gene expression data (probabilistic gene networks); iii - novel experiments considering binary and ternary genes (instead of only binary) and adopting single and multiple GPUs (one, two and four).

### Identifying predictors by probabilistic gene networks using mutual information

Expression profiles of *predictor genes *display relevant informative content (individual or in conjunction with other predictors) about the expression profile of a given *target gene*. Feature selection methods can be employed to find the subset of genes (predictors) presenting the largest information content about the target gene values.

We adopted the probabilistic genetic network (PGN) approach [11-13] which follows the feature selection principle: for each target, a search for the subset of predictors that best describes the target behavior according to their expression signals is performed. Barrera *et al*. discusses this approach in the context of the analysis of dynamical expression signals of the Plasmodium *falciparum *(one of the agents of the malaria disease), providing interesting biological results [11]. This approach assumes that the temporal samples follow a first order Markov chain in which each target gene value in a given instant of time depends only on its predictor values at the previous instant of time. The transition function is homogeneous (it is the same for every time step), almost deterministic (from any given state, there is one preferential state to go in the next time) and conditionally independent.

Lopes *et al*. [13] provides a comparative study involving this approach (using the Sequential Floating Forward Search as search algorithm) and methods like MRNET [8], ARACNE [9] and CLR [10]. Such approach showed superior performance for retrieving multivariate predictors. The mean conditional entropy (MCE), indicating the average information content of the target gene given its predictors, was adopted as fitness function. Mutual information is a measure of dependence between variables that has been employed in many research fields such as image processing [24,25], physics [26] and bioinformatics [27,28]. The main advantage of mutual information compared to other similarity measures such as Pearson correlation is the capability to capture non-linear relationships between variables [28].

The exhaustive search that looks for all possible pairs of candidate predictors for each target was considered as search method. In fact, it is the only way to obtain optimality in feature selection due to the intrinsically multivariate prediction, which may be present in biological systems [29]. Such phenomenon is related to the nesting effect that occurs when a greedy feature selection algorithm or other sub-optimal heuristics are applied. Once the exhaustive search is applied for all genes considered as target, the network is achieved.

### Search algorithm

Given a set *G *of genes, the search algorithm identifies, for each target gene *y *∈ *G*, the best subset *X *⊆ *G *that predicts *y *according to a criterion function. The following algorithm performs an exhaustive search in order to identify the pairs (X,y):

**Algorithm 1 **: ExhaustiveSearch

1: **for **each target gene *y *∈ *G ***do**

2:    **for **each predictor genes subset *X *⊆ *G ***do**

3:        calculates the criterion function *H *of the prediction of *y *by *X*

4:    **end for**

5: **end for**

### Criterion function

The mean conditional entropy (MCE) was adopted as criterion function. The Shannon's Entropy [30] of a variable *Y *is defined as

$$H\left(Y\right)=-\underset{y\in Y}{\Sigma }P\left(Y=y\right)\text{log}P\left(Y=y\right),$$

where *P*(*Y *= *y*) is the probability of the variable *Y *be equal to *y*. The conditional entropy of *Y *given *X *= *x *is:

$$H\left(Y|X=x\right)=-\underset{y\in Y}{\sum }P\left(Y=y|X=x\right)\text{log}P\left(Y=y|X=x\right),$$

where *X *is a feature vector and *P*(*Y *= *y*|*X *= *x*) is the conditional probability of *Y *be equal to *y *given the observation of an instance *x *∈ *X*.

Lastly, the *Mean Conditional Entropy *(MCE) is defined as the weighted average of the conditional entropies [11-13]:

$$H\left(Y|X\right)=\underset{x\in X}{\Sigma }P\left(X=x\right)H\left(Y|X=x\right).$$

## GPU architecture and CUDA

GPUs (*Graphics Processing Unit*) are programmable graphic processor which, in combination with CPUs, can be used as a general purpose programming platform. They are optimized to perform vector operations and floating point arithmetics, executing in SIMD (Simple Instruction, Multiple Data) mode [31].

**Figure 1:**
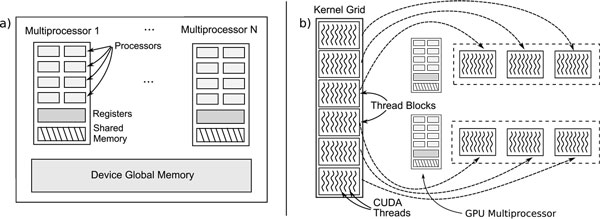
**The CUDA platform**. a) Architecture of a modern GPU containing a large global memory and a set of multiprocessors, each one with an array of floating-point processors, a small shared memory and a large number of registers. b) Hierarchical organization of CUDA threads in thread blocks and in kernel grids, where each thread block is assigned to a single multiprocessor.

Each GPU has a set of *Streaming Processors *(SMs), each constituted by an array of processor cores, which are the logical-arithmetic units of the GPU, as shown in Figure 1(a). Each SM has a large number of registers, a small control unit and a small amount of shared memory, accessible from the threads executing in the SM. Graphical devices normally have a large amount of global memory, which is shared among the SMs. The latency for accessing this memory is high and, consequently, the shared memory is normally used as a user controlled cached.

CUDA (*Compute Unified Device Architecture*) is a platform that provides an extension to the C language that enables the usage of GPUs as a general purpose computing device. A compiler generates executable code for the GPU device from the provided CUDA code.

The programmer defines special functions called *kernels*, which are executed in the GPU. The user defines the number of threads to create, organizing them in thread blocks. The collection of blocks from a single kernel execution is called *grid*. Each thread block runs on a single SM, but multiple blocks can be assigned to the same SM in a time-shared way. The CUDA programming model is shown in Figure 1(a).

## A GPU/CUDA algorithm for GRNs inference

The general concept of the parallel exhaustive search consists in distributing the fitness function computation along the SMs. The algorithm partitions the set of target genes *T *into *k *segments *T*_0_, *T*_1_, …, *T*_*k*−1 _and distributes these segments among the thread blocks. Each thread block is responsible for evaluating the criterion function for its assigned target genes *T_i_*for every pair of predictors from the set of genes *P*.

### Preliminary considerations and user settings

Given *G *with *n *genes, the complexity of the exhaustive search is *O*(*n*^|*X*|^) for each evaluated target, where |*X*| is the number of predictors. This occurs because for each target we must evaluate the entropy for every p-tuple of predictor genes. If every gene is used as target, we have a total complexity of *O*(*n*^*p*+1^).

For larger values of |*X*|, this procedure becomes impractical for typical values of *n *(thousands in microarray experiments). In this way, the number of predictors was fixed in two (|*X*| = 2) to reduce the search space. From the biological point of view, this decision is reasonable since the average number of predictors in GRN is between 2 and 3 according to some previous studies [32]. Besides, in a typical microarray experiment, only dozens of samples are available, which leads to a weak statistical estimation if one considers subsets with 3 or more predictors per target [11].

### Preprocessing

**Figure 2:**
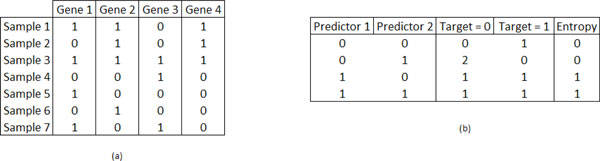
**Input expression matrix example**. a) Input expression matrix with 7 samples and 4 genes. Two copies of such matrix are assigned to matrices *T *and *P*. b) Evaluation of the mean conditional entropy based on the conditional probabilities of a target given two predictors.

Initially, the program reads the expression matrix from the disk and replicates it into two matrices *T *and *P *in the main memory. Matrices *T *and *P *represent the expressions of the target and candidate predictor genes, respectively. Each matrix has s lines, representing the experiment samples, and *n *columns, represent each gene. Figure 2(a) shows an example matrix with 4 genes and 7 samples. After loading the data into *T *and *P *into the main memory, the program allocates space and transfers the matrices to the GPU global memory.

### Local exhaustive search

**Figure 3:**
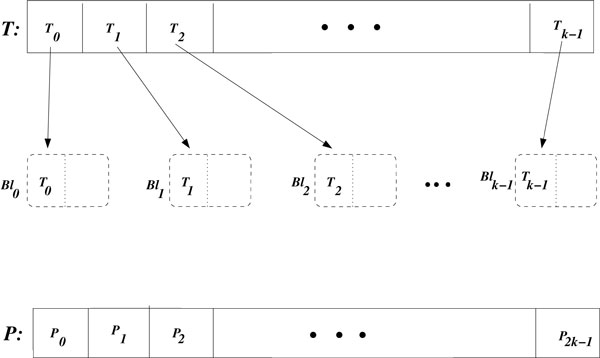
**Matrices partitioning**. Partitioning the matrices *T *and *P *in *k *segments and blocks access to segments of *T*: an arrow *T_i_*→ *Bl_i_*indicates that the block *Bl_i_*accesses the segment *T_i_*.

We consider that *k *blocks are started, denoted by *Bl*_0_, *Bl*_1_, …, *Bl*_*k*−1_. The algorithm then partitions the set of target genes *T *into *k *segments *T*_0_, *T*_1_, …, *T*_*k*−1 _of size *n*/*k *and the set of predictor genes *p *into 2*k *segments *P*_0_, *P*_1_, …, *P*_2*k*−1 _of size *n*/2*k*, as illustrated in Figure 3. Each thread block is responsible for evaluating the criterion function for its assigned target genes in *T_i_*for every pair of predictors from the set of genes *P*.

Each thread evaluates the conditional entropies, for every pair of predictors, of a single target gene in *T_i_*. To evaluate the entropies, each thread block transfers to the shared memory of its SMs parts of tables *T *and *P *containing the set of target genes *T_i_*and two sets of predictors *P*_*j*1_and *P*_*j*2_. These data are transferred from the global memory in a coalesced way, which joins up to 32 individuals memory reads into a single one, increasing the effective memory bandwidth.

Algorithm 2 describes the exhaustive search procedure executed by each block. To evaluate the conditional entropies of a target gene in *T_i_*for each pair of predictors in *P*_*j*1_and *P*_*j*2_, the thread creates a table, shown in Figure 2(b). This table contains the number of times a gene in *T_i_*assumed the values 0 or 1 for each combination of the predictor genes values, and the associated conditional entropy. The threads maintain this table at the registers of their associated SMs during the evaluation of the entropies, preventing expensive global memory accesses.

**Algorithm 2 **: LocalExhaustiveSearch

**Require: **segment *T_i _*of target genes and segments *P*_*j*1_and *P*_*j*2_of predictor candidates

1: **for **each target *t *∈ *T_i_***do**

2:    **for **each pair (*p*1, *p*2) ∈ {*P*_*j*1_× *P*_*j*2_} **do**

3:        calculates the power of (*p*1, *p*2) to predict *t *according to a criterion function *H*

4:    **end for**

5: **end for**

### Global exhaustive search

**Figure 4:**
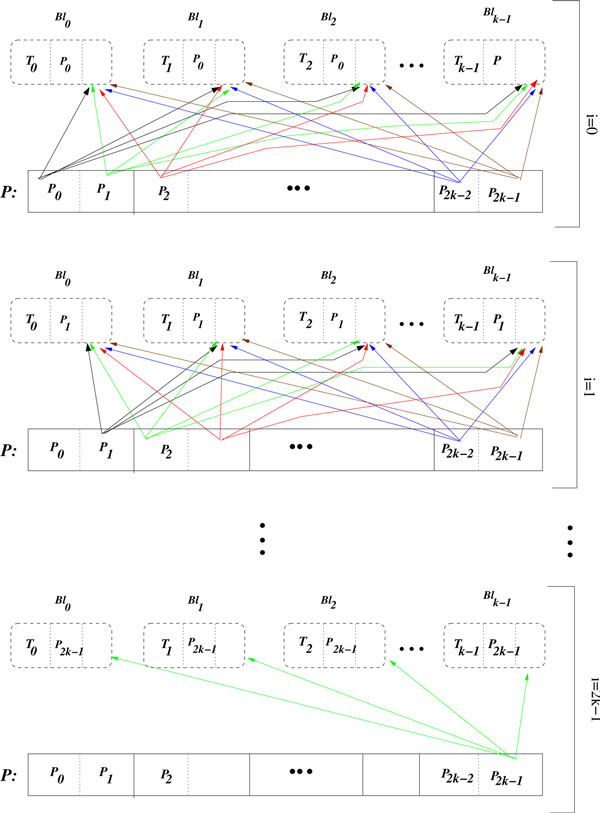
**Access rule of blocks to segments**. An arrow *P_i_*→ *Bl_j_*indicates that the block *Bl_j_*accesses the segment *P_i_*. Arrows of the same color indicate that the accesses are simultaneously performed.

The global exhaustive search provides, for each thread block, all pairwise combinations of predictor subsets *P*_*j*1_and *P*_*j*2_. With these permutations along the segments of *P*, each thread block can evaluate all predictor candidate pairs for every target gene in *T_i_*, as described in Algorithm 3 and illustrated in Figure 4.

**Algorithm 3 **: GlobalExhaustiveSearch

1: Transfers the table values for the genes belonging to *T_i_*

2: **for ***j*1 ← 0 to 2*k *− 1 **do**

3:    Transfers the table values for the genes belonging to *P*_*j*1 _to all blocks

4:    **for ***j*2 ← *j*1 to 2*k *− 1 **do**

5:        Transfers the table values for the genes belonging to *P*_*j*2 _to all blocks

6:        Evaluates the entropy for every pair of predictors (*p*1, *p*2) ∈ {*P*_*j*1_× *P*_*j*2_}

7:    **end for**

8:    **end for**

As we will analyze in the next section, this algorithm reduces the number of transfers from global memory for each predictor gene from set *P*. Moreover, it transfers each target gene from set *T *only a single time, in the beginning of the algorithm.

Besides reducing the number of global memory transfers for each gene, by dividing the tables into contiguous sets, we can perform coalesced transfers [31] from the global to the shared memory, further increasing the bandwidth of the memory. In this kind of transfer, up to *w *memory values are transferred as a single memory access. *w *is architecture dependent and has a value of 32 in the tested GPUs. Consequently, the algorithm works optimally for multiple of 32 genes, since the GPUs execute the threads in clusters (warps) of 32. Thus, the transfers between shared and global memories and the use of GPU cores are optimized. For different GRN sizes, *dummy *genes might be included to the GRN.

### Analysis of the algorithm

Considering a single thread block, for each iteration of the outer loop from Algorithm 3, one segment of genes *P*_*j*1 _is transferred from the memory. For the inner loop, there are 2*k *− *j*1 iterations for each value of *j*1, where on each iteration the segment *P*_*j*2 _is transfered. Consequently, the number of segment transfers per thread block, considering the inner and outer loops, is:

$$\overset{2*k-1}{\underset{j1=0}{\sum }}2*k-j1+1=2+3+\ldots +\left(2k+1\right)=2k^{2}+3k$$

We must add to this value the transfer of *T_i_*in the beginning of the algorithm. Considering that there are *k *thread blocks operating simultaneously and that there are *n*/2*k *genes per segment, the total number of gene transfers will be:

$$k*\frac{n}{2k}*\left(2k^{2}+3k+1\right)=n\times \left(k^{2}+\frac{3}{2}k+\frac{1}{2}\right)$$

Consequently, we can see that the number of gene transfers from the global memory is $O\left(n*k^{2}\right)$ and that each gene is transfered *O*(*k*^2^) times. This means that by increasing the segment sizes, we have a smaller *k *less transfers from the global memory.

Also, if the shared memory is not used, the total number of gene transfers would be *O*(*n*^3^), resulting in a memory load (*n*/*k*)^2 ^times higher. For *n *= 4096 predictors and segments of size 128, resulting in *k *= 32 blocks, the number of transfers without the segmentation would be 128 * 128 = 1.6 * 10^4 ^times higher. This difference occurs because when a segment is transferred to the shared memory, the values for each gene from the segment are used multiple times.

### Multi-GPU algorithm

In order to provide scalability for our method and improve its performance, we extended our inference algorithm to work with multiple GPUs. The general idea of the multi-GPU algorithm is to partition the set of target genes among the available GPUs and execute Algorithms 2 and 3 in each GPU. Consequently, each GPU is responsible for calculating the entropy of a subset of target genes.

Suppose we have *m *GPUs denoted by *C*^0^, *C*^1^, …, C*^m−1^*. The multi-GPU algorithm is described as follows:

1. Copy matrix *P *to the global memory of each GPU. Then, partition matrix *T *in *m supersegments *of size *n*/*m*, which we denote by *T*^0^, *T*^1^, …, *T^m−1 ^*(we assume here that *n *is a multiple of *m*). Copy each supersegment *T^i^*to the global memory of *C^i^*, 0 ≤ *i *≤ *m *− 1.

2. Launch the kernels with *k*/*m *thread blocks per GPU, where *k *is the total number of blocks. We denote by $Bl_{j}^{i}$ the block $Bl_{j}$ started in *C^i^*, where 0 ≤ *j *≤ *k*/*m *− 1 and 0 ≤ *i *≤ *m *− 1.

3. Execute Algorithms 2 and 3 on each GPU, so that GPU *C^i^*receives the blocks $Bl_{0}^{i},Bl_{1}^{i}\ldots ,Bl_{k/m-1}^{i}$, which operate on the segments $T_{0}^{i},T_{1}^{i}\ldots ,T_{n/m-1}^{i}$. Here $T_{j}^{i}$ denotes the segment *j *belonging to supersegment *i*.

4. Copy the best predictor pairs for each target gene along with their corresponding entropy values from the GPU global memory to the CPU main memory.

**Figure 5:**
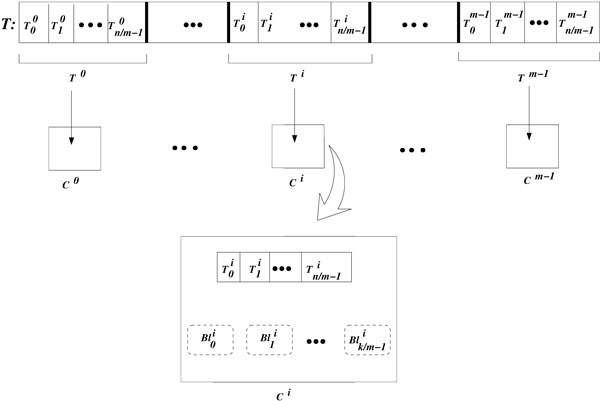
**Multi-GPU Algorithm**. An arrow *T^i^*→ *C^i^*indicates that the supersegment *T^i^*is copied to the global memory of GPU *C^i^*; $T_{j}^{i}$ denotes the segment *j *belonging to supersegment *i*; $Bl_{j}^{i}$ denotes the block *Bl_j_*started in *C^i^*.

Figure 5 shows a schematic representation of the main characteristics of the multi-GPU algorithm.

### Implementation

The implementation of the parallel exhaustive search algorithm was performed using CUDA. We applied all optimizations described in the algorithm description. The implementation source code can be obtained at https://sourceforge.net/p/inferencemgpu/.

The CPU implementation, which we used to evaluate the speedups, utilizes *OpenMP *to enable the usage of all cores from the processor. OpenMP is an API (*Application Program Interface*) designed for implementing parallel algorithms in architectures with shared memory multiprocessors. We divided the target genes among the threads, resulting in a good load-balancing among the cores.

## Results and discussion

We executed the CPU version of the algorithm in a machine with a six-core Intel i7 3930K 3.2 GHz processor and 32GB of DDR3 RAM memory. For the GPU implementation execution, we used a quad-core computer with Intel i7 920 2.6 GHz with 6 GB of DDR3 RAM memory and 2 NVIDIA GTX 295 graphic boards, with 2 GPUs and 1792 Mb of memory on each board. Each GPU has 30 multiprocessors (SMs) with 8 cores on each, resulting in a total of 240 cores per GPU. We used Linux Ubuntu 12.04, with CUDA version 4.2 and gcc 4.6.3 compiler, configured with the option -O3.

In both versions, we measured the elapsed times of the complete execution of the application. In the GPU version, this means that the time necessary to allocate the variables in the CPU and GPU memories and the data transfers are included in the measurements.

The binary data samples used in the experiments presented in this section were generated using the Artificial Gene Network (AGN) simulator [33], considering the Erdös-Rényi Boolean network model. Such simulator allows to control the number of genes present in the network and the number of samples. We also performed experiments considering ternary data samples, i.e., genes can be underexpressed, normal and overexpressed (-1, 0 +1). For this set of experiments, we considered the *Plasmodium falciparum *database [34], the same database considered in [11], whose expression values were quantized in three values by applying normal transform (Z-Score).

### Execution times for binary samples

We performed three experiments to evaluate the performance of our method, comparing the execution times of the CPU implementation with the GPU algorithm running in 1, 2 and 4 GPUs. We used datasets with 30 binary samples and GRNs with different sizes (1024, 2048, 4096, 8192).

Tables 1, 2 and 3 show the average execution times for each experiment (3 executions for each experiment) considering 32, 64 and 128 target genes per block. For a fixed GRN dize, larger number of target genes per block implicates in smaller execution times. This happens because the higher the number of target genes processed per block, the higher the number of genes processed in parallel in each block, which leads to less traffic between shared and global memories. Experiments with *targets/block *> 128 were not performed, since the GPU shared memory does not support the segment lengths of the expression matrices *T *and *P*. The small amount of shared memory is an important restriction of the GPU architecture.

**Table 1:** Experiment 1: Execution times for binary samples experiment using 1 GPU.

GRN size	CPU	32 targets/bl	64 targets/bl	128 targets/bl
1024	4 min	19.8s s	20.1 s	9.3 s
2048	34.5 min	2.7 min	1.4 min	36 s
4096	5 h	16.4 min	11 min	4.8 min
8192	1.7 days	1.8 h	1.1 h	28.6 min

Columns from left to right: different sizes of GRNs (number of genes); execution times of the 6 core CPU algorithm; execution times for 3 experiments using the GPU algorihm: 32, 64 and 128 target genes per block using 1 GPU.

**Table 2:** Experiment 2: Execution times for binary samples experiment using 2 GPUs.

GRN size	CPU	32 targets/bl	64 targets/bl	128 targets/bl
1024	4 min	19.5 s	20 s	9.2 s
2048	34.5 min	1.4 min	1.3 min	35 s
4096	5 h	13.3 min	5.6 min	2.4 min
8192	1.7 days	1.1 h	43 min	18.7 min

Columns from left to right: different sizes of GRNs (number of genes); execution times of the 6 core CPU algorithm; execution times for 3 experiments using the GPU algorihm: 32, 64 and 128 target genes per block using 2 GPUs.

**Table 3:** Experiment 3: Execution times for binary samples experiment using 4 GPUs.

GRN size	CPU	32 targets/bl	64 targets/bl	128 targets/bl
1024	4 min	19.5 s	20 s	9.3 s
2048	34.5 min	1.3 min	1.3 min	35 s
4096	5 h	5.4 min	5.4 min	2.3 min
8192	1.7 days	43 min	22.3 min	9.4 min

Columns from left to right: different sizes of GRNs (number of genes); execution times of the 6 core CPU algorithm; execution times for 3 experiments using the GPU algorihm: 32, 64 and 128 target genes per block using 4 GPUs.

We verified that as we increase the number of targets per block, the execution time decreased as expected, since the number of transfers from global memory for each gene is *O*(n * *k*^2^). But the number of operations for evaluating the entropies is the same, regardless of the number of targets per block. This explains the almost linear gains in performance when increasing the block size from 32 to 128 targets per block.

### Execution times for ternary samples

The same experiments performed for binary samples were also run considering 30 ternary samples (considering that each gene can assume three values). Tables 4, 5 and 6 show the average execution times for each experiment (3 executions for each experiment) considering 32, 64 and 128 target genes per block.

**Table 4:** Experiment 4: Execution times for ternary samples experiment using 1 GPU.

GRN size	CPU	32 targets/bl	64 targets/bl	128 targets/bl
1024	4.2 min	48.5 s	28.7 s	13.5 s
2048	36.9 min	5 min	3.9 min	53 s
4096	5.6 h	33.8 min	24.5 min	7.4 min
8192	1.9 days	4.7 h	2.7 h	45.3 min

Columns from left to right: different sizes of GRNs (number of genes); execution times of the 6 core CPU algorithm; execution times for 3 experiments using the GPU algorihm: 32, 64 and 128 target genes per block using 1 GPU.

**Table 5:** Experiment 5: Execution times for ternary samples experiment using 2 GPUs.

GRN size	CPU	32 targets/bl	64 targets/bl	128 targets/bl
1024	4.2 min	24.5 s	28 s	13.1 s
2048	36.9 min	3.3 s	1.9 min	50 s
4096	5.6 h	20.2 min	16 min	3.7 min
8192	1.9 days	2.7 h	1.6 h	29 min

Columns from left to right: different sizes of GRNs (number of genes); execution times of the 6 core CPU algorithm; execution times for 3 experiments using the GPU algorihm: 32, 64 and 128 target genes per block using 2 GPUs.

**Table 6:** Experiment 6: Execution times for ternary samples experiment using 4 GPUs.

GRN size	CPU	32 targets/bl	64 targets/bl	128 targets/bl
1024	4.2 min	24 s	28 s	12 s
2048	36.9 min	1.7 min	1.9 min	49 s
4096	5.6 h	13.3 min	8 min	3.5 min
8192	1.9 days	1.6 h	1.1 h	14.5 min

Columns from left to right: different sizes of GRNs (number of genes); execution times of the 6 core CPU algorithm; execution times for 3 experiments using the GPU algorihm: 32, 64 and 128 target genes per block using 4 GPUs.

The same observations stated for the binary samples case are also valid here.

### Obtained speedups

**Figure 6:**
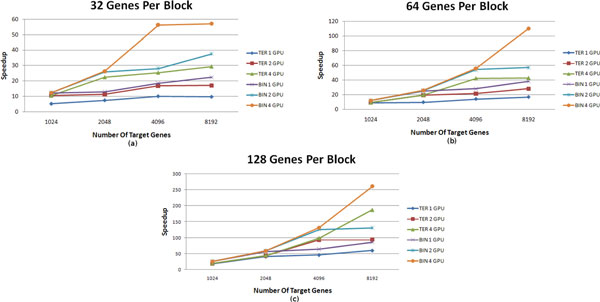
**Speedup × GRN size**. (a), (b) and (c): graphics representing speedup × GRN size for 32, 64 and 128 targets per block. The terms "BIN" and "TER" indicate binary and ternary samples cases, respectively.

We also evaluated the speedups obtained with the GPU algorithm. We defined the speedup as the execution time spent by the multi-core algorithm parallelized on 6 CPU cores divided by the execution time spent by the GPU algorithm for the same instance of the problem. Figures 6(a), 6(b) and 6(c) show the speedup *versus *number of genes considering 32, 64 and 128 target genes per block, respectively. These results consider both binary and ternary samples.

The results show good speedups on networks with 2048 or more genes, especially when using two or four GPUs. For example, using four GPUs for networks of 8192 genes, we obtain speedups of approximately 55, 110 and 260, when using 32, 64 and 128 target genes per block, respectively, for the binary samples case. For ternary samples, the speedup behaviors look similar, with speedups of approximately 30, 40 and 185 for 32, 64 and 128 targets per block, respectively. Moreover, the speedup tends to increase with the number of genes, since in this case we use all the cores in the GPUs more effectively.

The speedup obtained with the ternary samples was smaller than with the binary samples because each thread uses more state variables. This results in a larger register utilization and, consequently, a smaller number of simultaneously executing threads. However, the obtained speedup is 185 when using 4 GPUs and 60 when using a single GPU.

Regarding the usage of multiple GPUs, Figure 6(a) shows that, for the binary coding, there is no advantage in using two or four GPUs when we take 32 target genes per block and GRNs with 1024 genes. With ternary coding, the execution times with two and four GPUs were the same. A similar scenario occurs for networks with up to 2048 genes and 64 and 128 genes per block, as shown in Figure 6(b) and 6(c).

This result can be explained considering the number of thread blocks required to represent all target genes. For instance, considering 32 targets per block, we need 32, 64 and 128 blocks for networks with 1024, 2048 and 4096 genes, respectively. In the experiments we used GPUs with 30 SMs, which can simultaneously execute a number of blocks multiple of 30. With 32 genes per block and 1024 genes, the speedups with 1, 2 and 4 GPUs were the same. In this case, it is clear that with one GPU, 2 SMs executed 2 blocks simultaneously, with the others executing a single block, without a performance penalty. With 2048 genes there are 64 blocks and there was a performance gain when using 2 or more GPUs. In this case, with one GPU some SMs had to execute three blocks, which could not be performed simultaneously. Consequently, it required almost twice the time when compared to the execution with 2 GPUs.

Finally, for networks with larger number of genes, such as 8192 genes, the use of multiple GPUs provides important gains in the speedup. For instance, considering 128 genes per block using 4 GPUs (Figure 6(c)), the speedup was 2 times higher than the obtained by considering 2 GPUs and 3 times higher than the obtained by considering a single GPU when applying to binary samples. And in the ternary samples case, considering the same settings, the speedup obtained for 4 GPUs was 2 times higher than the obtained by considering 2 GPUs and 3.1 times higher than the obtained by considering a single GPU.

### Number of samples

**Figure 7:**
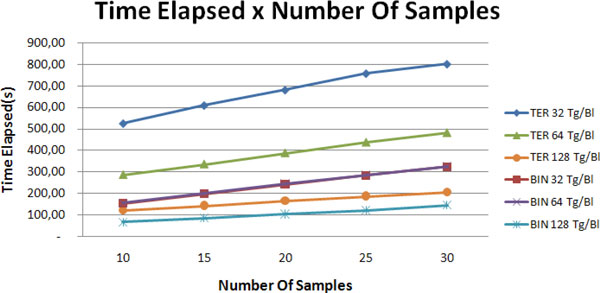
**Time elapsed in function of the number of samples**. Curves representing time elapsed in function of the number of samples, in which each curve corresponds to a number of targets per block (32, 64 and 128). In this case, 4 GPUs were employed to infer a network of 4096 genes. The terms "BIN" and "TER" indicate binary and ternary samples cases, respectively.

To evaluate the dependence of the runtime on the number of samples, we conducted an experiment using four GPUs and a network with 4096 genes. We varied the amount of samples and target genes per block. The results (see Figure 7) show that there is a linear dependence between the runtime and the number of samples for both binary and ternary samples cases.

## Conclusions

In this paper we propose a multi-GPU algorithm that allows the inference of gene regulatory networks (GRNs) with multivariate predictions in significantly lower times than using multi-core CPUs. For instance, the inference of a GRN with 8192 genes, which took about two days in a six core CPU, was executed in less than 30 minutes using 1 GPU and about 10 minutes using 4 GPUs. The main contribution of the algorithm is to permit the execution of the exhaustive GRN inference method using large datasets in a reasonable time.

Another important observation is that the proposed multi-GPU scheme is well scalable, since the speedups increased in an almost linear fashion with the employed number of GPUs. Such speedups results suggest that it is an efficient and low cost solution for researchers that need to infer GRNs of realistic sizes (order of thousands) from transcriptome data in a reasonable time, considering multivariate (N-to-1) relationships. Besides, this paper presents a proof of principle, showing that it is possible to parallelize the exhaustive search algorithm in GPUs with encouraging results. Although our focus was on the GRN inference problem, we developed an exhaustive search technique based on GPU which can be applied to other combinatorial problems with minor adaptations.

As future work, the algorithm will be improved to work with predictor subsets with cardinality greater than 2, which allows to infer GRNs with more complex interactions. Such improvement requires new approaches for gene expression matrices division and for data traffic management between the global and shared memories. Also, we will also update the method to execute in clusters of heterogeneous GPUs, which will provide more performance, specially for inferences with larger networks and higher cardinalities.

## Competing interests

The authors declare that they have no competing interests.

## Authors' contributions

All authors analyzed the initial problem, conceived the general framework of the proposed approach and discussed aspects of the development and implementation. FFB and RYC worked on development and implementation of the method. All authors idealized the experiments, discussed the results and participated in the production and revision of the manuscript.

## Declarations

Publication of this article was supported by Conselho Nacional de Desenvolvimento Científico e Tecnológico (CNPq proc. 559955/2010-3).

This article has been published as part of *BMC Bioinformatics *Volume 14 Supplement 18, 2013: Selected articles from the Second IEEE International Conference on Computational Advances in Bio and Medical Sciences (ICCABS 2012): Bioinformatics. The full contents of the supplement are available online at http://www.biomedcentral.com/bmcbioinformatics/supplements/14/S18.
